# A proposal for a new PhD level curriculum on quantitative methods for drug development

**DOI:** 10.1002/pst.1873

**Published:** 2018-07-09

**Authors:** T. Jaki, A. Gordon, P. Forster, L. Bijnens, B. Bornkamp, W. Brannath, R. Fontana, M. Gasparini, L.V. Hampson, T. Jacobs, B. Jones, X. Paoletti, M. Posch, A. Titman, R. Vonk, F. Koenig

**Affiliations:** ^1^ Lancaster University Department of Mathematics and Statistics Lancaster UK; ^2^ Janssen Pharmaceutica NV Beerse Belgium; ^3^ Novartis Pharma AG Basel Switzerland; ^4^ University of Bremen KKSB and IfS Faculty 3 – Mathematics/Computer Science Bremen Germany; ^5^ Politecnico di Torino Turin Italy; ^6^ INSERM CESP‐OncoStat Institut Gustave Roussy & Université Paris‐Saclay UVSQ & Service de Biostatistique et d'Epidémiologie Gustave Roussy Villejuif France; ^7^ Medical University of Vienna Center for Medical Statistics, Informatics, and Intelligent Systems Vienna Austria; ^8^ Bayer AG Berlin Germany

**Keywords:** development of early‐stage researchers, drug development, PhD curriculum, regulatory statistics, university‐industry partnership

## Abstract

This paper provides an overview of “Improving Design, Evaluation and Analysis of early drug development Studies” (IDEAS), a European Commission–funded network bringing together leading academic institutions and small‐ to large‐sized pharmaceutical companies to train a cohort of graduate‐level medical statisticians. The network is composed of a diverse mix of public and private sector partners spread across Europe, which will host 14 early‐stage researchers for 36 months. IDEAS training activities are composed of a well‐rounded mixture of specialist methodological components and generic transferable skills. Particular attention is paid to fostering collaborations between researchers and supervisors, which span academia and the private sector. Within this paper, we review existing medical statistics programmes (MSc and PhD) and highlight the training they provide on skills relevant to drug development. Motivated by this review and our experiences with the IDEAS project, we propose a concept for a joint, harmonised European PhD programme to train statisticians in quantitative methods for drug development.

## 1 INTRODUCTION

Drug development is a long and costly process. This, paired with the high levels of attrition seen even at late stages of the process, has increased the emphasis being placed on quantitative decision‐making during the biopharmaceutical development process. More quantitative assessment of risk is now performed when moving from early‐ to late‐stage clinical trials, for example. To avoid lengthy and expensive confirmatory studies, the use of efficient methods for the design and analysis of early‐phase clinical studies is essential. The challenges faced by statisticians working in this field^1^, ^2^, ^3^ range from model specification,^4^ small sample sizes,^5^, ^6^ and complex in silico simulations, to how relevant information can be translated and used. In recent years, demand for quantitative skills from both academia and industry has surged due to greater emphasis being placed on quantitative benefit‐risk assessment, dose‐finding, extrapolation, and the use of related data. For example, there are now several initiatives allowing access to individual patient data,^7^ which have resulted in the wider uptake of Bayesian methods as this facilitates the use of informative priors and innovative clinical trial designs^8^, ^9^ to directly incorporate available information in decision‐making. One of the challenges with this more quantitative approach is that expertise in these methods is limited; this is in part due to them being quite specialised so that they are not routinely covered in standard educational programmes. Another important facet of a statistician's work is that they collaborate with multidisciplinary teams, another aspect not captured by current training programmes. Although it is explicitly stated in ICH guidelines E6^10^ and E9^11^ that a statistician must be involved in the conduct of a clinical trial and the multiple demands on a statistician in the pharmaceutical industry have been discussed in Chuang‐Stein et al,^12^ there is no unique definition of what core training a statistician should receive. Despite the lack of unique definition, a survey by the European Federation of Statistician in the Pharmaceutical Industry in 1999 already shows that a notable portion of statisticians (10%‐40%) in the European industry has a PhD.^13^ A follow‐on survey undertaken among IDEAS partners shows that this has risen to at least 30% by 2018 and in some companies and regions is well over 50%.

In this paper, we will describe the approach taken by the IDEAS network on “Improving Design, Evaluation and Analysis of early drug development Studies” (http://www.ideas-itn.eu) funded by the European Commission (EC) under the Marie Sklodowska‐Curie Actions to overcome this challenge to educate world‐class PhD‐level researchers. We argue that an expanded pan‐European curriculum is the logical next step towards ensuring a continued supply of well‐educated statisticians who are prepared for careers in the pharmaceutical industry, regulatory agencies, or public sector medical research organisations. In Section 2, we explore the training typically offered by relevant MSc programmes (eg, MSc in [Medical] Statistics and MSc in Biometry). We then highlight the specific requirements of current European PhD schemes in Section 3 and subsequently summarise other relevant formal accreditations available from learned societies (Section 4). In Section 5, we describe the IDEAS approach, focusing on the added value of the programme before we state our case for the development of a harmonised curriculum and critically evaluate the impact and risk of such a venture (Section 6).

## 2 EXISTING MSc PROGRAMMES

The natural start for someone aspiring to work as a statistician in drug development in Europe is to undertake an MSc in (Medical) Statistics. The typical training elements of such degree schemes are highlighted in Table 1. Because of space and language constraints, we limit this illustration to countries IDEAS partners are affiliated with. Typically full‐time MSc studies last between 1 and 2 years and comprise a set of compulsory and optional courses that culminate in a master's thesis.

**Table 1:** Key aspects of MSc programmes in Europe on the example of countries affiliated with IDEAS

Country	Institution	Duration and Credits	Area of Study	Credits for		
				Thesis	Compulsory Modules	Elective Modules
Austria	http://slw.univie.ac.at/studieren/studienangebot/masterstudien/statistik-master/	2 y (120 credits)	Master's in Statistics	20	90	10
Belgium	https://www.uhasselt.be/studyguide?n=3&i=001	2 y (120 credits)	Master's in Statistics	24	89	7
Denmark	http://studies.ku.dk/masters/statistics/	2 y (120 credits)	MSc Statistics	30	30	60
France	http://www.master.ufrmath.upmc.fr/	2 y (120 credits)	Msc in Mathematics and applications	18	36	66
Germany	https://www.statistik.tu-dortmund.de/fileadmin/user_upload/Studium/Ordnungen/Pruefungs-_und_Studienordnungen/Pruefungsordnung_Master_Statistik_2015.pdf	2 y (120 credits)		30	40	50
Germany	https://www.dbs.uni-bremen.de/studienangebot/studien-berufsfelder/natur-umwelt/detail/study/medical-biometry-biostatistics-master-1/	2 y (120 credits)	MSc Medical Biometry/Biostatistics	30	82	8
Greece	http://biostatistics.med.uoa.gr/index.php/study-guide/	2 y (120 credits)	MSc Biostatistics	30	62	28
Ireland	https://sisweb.ucd.ie/usis/!W_HU_MENU.P_PUBLISH?p_tag=PROG&MAJR=T020	1 y (90 credits)	MSc Statistics	25	12.5	52.5
Italy	http://corsi.unibo.it/2Cycle/StatisticalSciences/Pages/course-structure-and-teaching-activities.aspx	2 y (120 credits)	MSc Statistical Sciences	30	80	10
Poland	http://wmat.pwr.edu.pl/studenci/studia/programy-studiow/studia-ii-stopnia/matematyka	2 y (120 credits)	Master's in Mathematical Statistics	18	49	53
Spain	http://www.usc.es/masteres/en/masters/science/statistical-techniques	18 mo (90 credits)	Master's in Statistical Techniques	10	30	50
Switzerland	https://www.unige.ch/gsem/en/programs/masters/statistics/	18 mo (90 credits)	MSc Statistics	30	33	27
United Kingdom	http://www.lancaster.ac.uk/maths/	12 mo (180 credits)	MSc in Statistics	60	70	50

A more detailed overview of the taught courses in the European MSc programmes above and all relevant Msc programmes in the UK is provided in Supporting Information (Tables S1 and S2). We list the compulsory modules in full for each programme but only include those optional courses that are particularly relevant to someone seeking to work with clinical trials. The compulsory modules for a large part focus on the fundamentals of statistics, such as inference, Bayesian methods, and computational statistics, while optional courses include “Introduction to clinical trials” and “Methods for analysing genetic data.” There is a lack of more specialised courses. For example, except for Lancaster University and the University of Dortmund, none of the programmes offer courses on adaptive methods or dose‐finding—topics highly relevant to drug development and at the forefront of ideas to improve the efficiency of clinical trials. As pointed out by one reviewer, there is only a limited amount of space in an MSc curriculum available and many competing topics. Such highly specialist modules at a level suitable for an MSc require lecturers who know the topic well, which demonstrates that our PhD programme would also address the academic need for experts in certain fields.

It is also notable that none of the programmes explicitly cites training in transferable skills as part of their curriculum. Although some aspects such as consultancy and presentation skills will likely be covered at most institutions as part of the offered courses, the distinct lack of explicit training indicates that this is not a highly valued part of the curriculum.

During the masters' thesis component of the masters' programmes (which in UK institutions will be around 3 mo in length but can be substantially longer at non‐UK institutions—in France, it usually lasts around 6 mo), focus is on one specific research question while methods and results are recorded in a report of 30 to 50 pages in length. Topics include theoretical/simulation–based as well as data‐based investigations. Pharmaceutical companies can either contribute topics and cosupervise students or occasionally host students subject to approval by the university. Clearly, opportunities exist for projects with industry, but in our experience, these are the exception rather than the norm.

As a result of the content and structure of these programmes, graduates can be expected to have a solid grounding in the foundations of statistics. Beyond that, however, they will have had limited training in the additional skills required for drug development.

## 3 FURTHER TRAINING THROUGH A PhD

One path often taken by junior researchers on their way to employment in drug development (eg, in industry, in a regulatory agency, in a contract research organisation [CRO], or in an academic clinical trials unit) is to follow their master's degree with a PhD. Many different PhD programmes exist, but again, the broad structure of these degrees is quite similar. The focus is on pursuing a research question. Although the question is often developed in conjunction with their supervisors, the student's work should be undertaken independently with some guidance by the supervisor. Despite some dedicated initiatives from public sector funders through cofunded studentships (eg, https://www.mrc.ac.uk/skills-careers/studentships/how-we-fund-studentships/industrial-case-studentships/) where students are hosted at academic institutions, or arrangements where the main basis of work can be within an industry partner (eg, http://www.iwt.be/english/funding/subsidy/BM), joint PhD projects with industry remain, however, the exception. The MRC CASE studentships, for example, only make up around 15% of MRC‐funded PhD places.

While most doctoral programmes in Statistics in Europe have the expectation that the student should also undertake further training beyond the research project, the extent to which this training is compulsory is often limited.

Table 2 summarises the formal requirements and further training expectations for universities that are part of the IDEAS network. While we do not claim that this is an exhaustive list of relevant PhD schemes, it does serve as a reference of what is considered typical for a PhD in Statistics (or related field) within Europe.

**Table 2:** Summary of PhD requirements of universities enrolling students on the IDEAS project

Institution	Further Training	Completion Requirement
Lancaster University	Expectation of 10 training days per year, which includes courses, seminars, workshops, and conferences	– Min 3 y; – PhD thesis containing significant novel research.
Medical University of Vienna	Several specific courses ranging from biomedical propaedeutics to journal clubs worth 34 ECTS credits	– Min 3 y (total 180 ECTS); – PhD thesis containing significant novel research (146 ECTS); – Completion of specific set of courses worth 34 ECTS credits.
Politecnico Di Torino	Twenty‐four ECTS worth of courses and workshops that can be freely chosen. At least 12 ECTS must come from lectures.	– Min 3 y; – PhD thesis containing significant novel research; – Completion of 24 ECTS credits worth of courses and workshops and attendance to seminars; – PhD awarded as “PD. in Mathematical Engineering”; – Short presentation of work when passing from years 1 to 2 and from years 2 to 3.
Universität Bremen	Expectation to undertake training in scientific writing, presentation skills, ….	– Min 3 y; – PhD thesis containing significant novel research.
University Paris Saclay	Thirty credits required over duration of studies. One credit is equivalent to 1 training day.	– Min 3 years; – PhD thesis containing significant novel research; – Completion of 30 training credits; – Two publications as first author.
Hasselt University	A set of courses (approximately 50 h in total) is to be attended.	– Min 4 y; – PhD thesis containing significant novel research.
Technische Universität Dortmund	Further optional training available, via either the statistics courses in the master program or specific courses on different nonstatistics topics, attendance of external trainings, workshops, and conferences.	– PhD thesis containing significant novel research; – Twenty ECTS credits worth of courses to be chosen from specific courses in the master's programme – Attendance of 20 departmental seminars – Two presentations in departmental PhD student seminar (at the beginning and the end of the PhD time)

From this small snapshot, we find that programmes at least have an expectation of some additional training beyond the main project of the PhD, with a substantial number requiring at least some formal training. Some institutions offer specific courses for PhD students, while others leave it up to the student/supervisor to decide what training is most important and appropriate. It is, however, notable that in all cases, this additional training only forms a small part (between 5% and 19%) of the degree scheme. It is also noteworthy that, with few exceptions, only subject matter training is deemed important; training on generic or transferable skills is often not explicitly required.

With this background, a new PhD graduate will be highly knowledgeable about their specific area of research but will often lack a broader awareness of the drug development process beyond the basics taught during their master's programme. Some experience with industry is possible for students who have been fortunate enough to work on a joint project with industry or have had the opportunity to undertake an internship at a company.

## 4 PROFESSIONAL QUALIFICATIONS FROM LEARNED STATISTICAL SOCIETIES

Although the involvement of a qualified statistician in drug development is an explicit requirement of ICH E6 and E9,^10^, ^11^ there is no unique definition of which education or what certificates from learned societies are sufficient. Some proposals have been published^14^, ^15^ and some formal certifications to qualify statisticians exist (Table 3), but consensus appears to be lacking. General certifications are, for example, available from the Royal Statistical Society and the American Statistical Association. However, only the German Society for Medical Computer Science, Biometry, and Epidemiology offers, together with the German Region of the International Biometric Society (IBS‐DR), a certification for Statisticians in Medicine.

**Table 3:** Professional certifications for statistician from professional societies

Organisation (Certificate)	Requirements	Comments
http://gmds.de/) together with the German Region of the International Biometric Society (IBS‐DR)	– A German university degree in medicine; statistics; or natural, social, or health sciences (at least to master's level) or a Master of Public Health or Epidemiology. – At least 5 y of experience in a relevant field. – A theoretical training that complements the input studying the complementary areas of expertise.	Three types of certification, namely, “Medical Computer Science,” “Biometrics in Medicine,” and “Epidemiology” depending on the area of work.
https://www.rss.org.uk/) Graduate Statistician	– A UK honours degree (class I or II) or equivalent overseas degree in statistics or related field. – Requires revalidation.	Nothing specific to medical statistics (or drug development)
https://www.rss.org.uk/‐chartered statistician	– As above. – Plus at least 5 y approved professional training and experience	Nothing specific to medical statistics (or drug development)
http://www.amstat.org/) graduate statistician (gstat)	– Advanced degree in statistics or a related quantitative field.	Nothing specific to medical statistics (or drug development)
http://www.amstat.org/) Accredited professional statistician (pstat)	– Advanced degree in statistics or a related quantitative field – At least 5 y of documented experience in the employment of appropriate statistical concepts and techniques – Required revalidation	Nothing specific to medical statistics (or drug development)
http://www.statsoc.org.au/ Accredited statistician (AStat)	– A degree with a statistics component equivalent to that of second‐ or third‐level statistics subjects or mathematics majors in Australian universities, plus 6 y practical experience in applying statistics; or a first‐ or second‐class honours degree or equivalent in statistics or in a subject containing substantial coverage of statistical methods or theory, plus 4 y practical experience in applying statistics.	Nothing specific to medical statistics (or drug development)
http://www.statsoc.org.au/ Graduate statistician (GStat)	– Fulfil the same degree requirement as for AStat, but completion date less than 8 y ago.	Nothing specific to medical statistics (or drug development)
https://ssc.ca/en Associate statistician (AStat)	– Completion of a course of study showing ability and aptitude in statistics (eg, an undergraduate degree in Statistics), or, in exceptional instances, has otherwise demonstrated an advanced understanding of statistical theory and its application.	Nothing specific to medical statistics (or drug development)
https://ssc.ca/en Professional statistician (PStat)	– As for AStat above – A minimum of 6 y of professional experience in the application of statistics	Nothing specific to medical statistics (or drug development)

All accreditations build upon a relevant university degree and have additional requirements with respect to the extent of practical experience. Additionally, further continued professional development is typically required, although there is no explicit requirement that this should incorporate elements of transferable skills training.

## 5 THE IDEAS APPROACH TO TRAINING

In previous sections, we have provided an overview of the conventional training paths available to statisticians and highlighted what we see as deficiencies in the training opportunities available to those interested in a career in drug development. These shortcomings are what motivated us to create the IDEAS network.

The network is composed of 8 partners (5 industrial and 3 academic) from 7 European countries (Austria, Belgium, France, Germany, Italy, Switzerland, and United Kingdom) that host one or more early‐stage researchers (ESRs) and is supplemented by 8 associated partners (5 industry and 2 academic) that contribute to the training activities of the network. The network is funded by the EC under the Marie Sklodowska‐Curie Actions. The funding covers salary of the ESRs and training costs (including costs of secondments) as well as management costs.

The primary objective of the network is train ESRs so that they are well equipped to work in drug development and have a good grasp of methods for studies in the early stages of drug development. Prior to joining, the 14 network ESRs have, with one exception, completed a master's degree in Statistics or related field (one student started with a BSc and had completed the first year of a 2‐year master's). The funding application to the EC states specifically that the network seeks to

- train ESRs in state‐of‐the‐art methods for designing, evaluating, and analysing early phase studies;
- develop novel methodology for early‐phase studies through individually supervised, collaborative, research projects;
- provide an international, collaborative environment, in which the academic research experience is paired with the challenges of undertaking drug development within the private sector; and
- raise awareness about cutting‐edge methods for designing and analysing early‐phase studies among statisticians, trialists, and clinicians alike.

In line with the rest of the manuscript, we will focus on the training of the ESRs (who are completing a PhD in Statistics or related field as part of their network research) and particularly highlight the novel approach taken. Note that some elements of what is implemented in IDEAS are also offered as part of other training programmes: The combination of these elements as well as the focus on early drug development is, however, unique. It should also be noted that training activities offered by the network are supplementing local requirements (ie, the university‐specific PhD curriculum) rather than superseding them. In some cases, aspects of the IDEAS training are counted as part of the local requirements (for example, training days for PhD students at Lancaster University). Day‐to‐day management of the network is undertaken by key representatives of each partner institution, along with 2 delegates representing the ESRs. Furthermore, management is overseen by an external advisory board composed of representatives from academia, industry, regulatory agencies, and clinical experts.

Traditionally training for medical statisticians is undertaken at academic institutions and at the MSc level both more theoretical and more practical degrees are offered. Interactions with the private sector or other stakeholders with an interest in applied problems are, however, far from the norm. The IDEAS network facilitates closer interactions between academia and the private sector while keeping the needs of clinical colleagues and regulators in sight. To achieve this, IDEAS ESRs who are based primarily at academic institutions spend secondments in industry and vice versa.

Training activities within the network, detailed below, comprise 4 components:

- individually supervised research projects;
- transnational, cross‐sectorial secondments;
- network‐wide training activities; and
- individual training activities.

### 5.1 Individually supervised research projects

The individual research project is, as with all other PhD schemes, the core of the activity of the ESRs. The IDEAS network does, however, take a collaborative approach to this activity so that cross‐sectorial cross‐disciplinary teams supervise the researchers. Each researcher is supervised by a primary contact at their host institution, as is typical for PhD research projects. In addition, a second supervisor also works with the student. The set‐up within IDEAS is such that the primary and secondary supervisors are based on complementary sectors so that a researcher at a university has a secondary supervisor from within industry and vice versa. This set‐up helps to give the ESRs greater experience of different research environments.

Additionally, supervisor pairings are transnational to allow the ESRs to see beyond national requirements and traditions. To supplement the methodological aspects of the research project, a clinical advisor is associated with each project. The role of this advisor, who is a highly respected researcher in a clinical area relevant to the project and often has additional experience with regulatory agencies, is to provide high‐level input on the direction and practicability of the project, provide the researcher with challenging problems from his subject matter field, and support implementation of the methods when appropriate. For example, in a project on Biosimilars,^16^ a review on experiences with EU approvals^17^ was conducted together with the associated clinical advisor.

### 5.2 Transnational, cross‐sectorial secondments

To further strengthen links between industry and academia and deepen collaborations between supervisory teams, each ESR will undertake one or more secondments. These transnational, cross‐sectorial secondments are visits to a partner in the complementary sector of at least 3 months' duration. The primary secondment is typically to the second supervisor's institution although more than one secondment is common. One of the main objectives of these visits is to expose the ESR to a different environment. The researchers will thereby gain insights into different host facilities, research, and training approaches beyond sectorial and national boundaries. The ESR works on problems related to their research (eg, a student looking a dose‐finding for combination studies might work on a project on combining safety and efficacy data in a single‐agent study) ideally resulting in joint publications.^18^, ^19^, ^20^, ^21^, ^22^ In addition, the ESR will also have the opportunity to get involved in the routine activities of a pharmaceutical statistician when seconded to an industry partner and will be able to contribute to consulting and teaching activities during academic placements. This arrangement also facilitates exchanges between the IDEAS partners beyond their specific PhD projects and enables the ESRs to establish a valuable professional network, which will benefit their future careers.

### 5.3 Network‐wide training activities

Compulsory network‐wide training sessions feature courses on relevant statistical methods specific to drug development as well as training on transferable skills. In addition, these events are also used to foster team building and joint research. Network‐wide activities are structured as

- four 5‐day summer schools;
- three e‐learning courses in statistical methodology;
- one Think Tank meeting; and
- regular interactive virtual discussion sessions run by and for the ESRs.

The objectives of the summer schools are to provide the ESRs with the relevant statistical skill set alongside more generic, transferable skills that are essential for any statistician working in medical statistics and in the early phases of drug development in particular. Additionally, these events include strategies to strengthen network‐wide interactions. See Table A1 for the complete summer school curriculum.

The e‐learning courses (detailed in Table A2) provide specialist courses in statistical methodology and computing, allowing the researchers to learn at their own pace, while ensuring personal interaction through online chat sessions and a discussion forum. The content of some e‐learning courses is subsequently reinforced in practical sessions at the summer schools.

The Think Tank meeting is held to ensure that the synergies between the different researchers can be used and provides the ESRs with an opportunity to experience and benefit from a cross‐sectorial collaborative environment.

The final components of training are virtual interactive discussion sessions. These sessions are ESR‐led virtual meetings held every 2 to 3 weeks that allow the researchers to discuss among themselves their projects and the challenges they are encountering. For many of the sessions, an ESR will present his/her current work, thereby developing their presentation skills while also providing them with the opportunity to receive constructive feedback on their research. The virtual sessions are also used to present and discuss individual research projects with the external advisory board of the IDEAS network, which is composed of one statistician in academia, a statistician working in industry, a member from a regulatory agency, and an academic clinician. Other sessions invite experts to provide tailored training that the students feel they need. For example, a session on “outreach” has been identified in this way.

### 5.4 Training activities tailored to the individual researcher

The final training component is individual training activities. These activities are established with emphasis on the specific needs of the individual ESR. The exact training programme is developed and agreed with the supervisory team and forms the basis of a personal career development plan. The individual training activities may range from specialised methodology training to career planning sessions to conference attendance. To complement the programme of short courses available at each partner site, the professional development courses offered at http://www.lancaster.ac.uk/maths/postgraduate/short-courses-and-cpd/, the https://www.meduniwien.ac.at/web/en/about-us/organisation/service-departments-and-staff-units/personal-und-personalentwicklung/, and the http://www.uni-bremen.de/career-center/veranstaltungsprogramm.html have been made available to all ESRs in the network.

## 6 ADDED VALUE OF A JOINT CURRICULUM IN QUANTITATIVE METHODS FOR DRUG DEVELOPMENT

In the previous section, we outlined the approach that the IDEAS network has taken to deliver a state‐of‐the‐art doctoral training programme for researchers interested in the statistical issues of the early phases of drug development. We believe that this initiative should be the first step towards ensuring an adequate pipeline of well‐trained researchers who can work and lead in the statistics of drug development more broadly. Progressing this idea, we believe that the next logical step is to establish a dedicated PhD‐level scheme, which can be run across several academic institutions jointly with the pharmaceutical industry and regulatory authorities and broadening the scope to other phases of drug development. The pharmaceutical industry and regulatory agencies will contribute by (co)supervising students on projects of interest to the organisation, teach on the training programme, and provide secondment opportunities. Emerging topics such as data science and big data to analyse and integrate data from various sources, eg, big data from clinical trials as well as real‐world evidence (RWE) generated, for example, from registries and health care providers^23^, ^24^ or new data sources such as Medical Internet of Things (eg, remote monitoring of patients with electronic devices),^25^ will change the way in which evidence is gathered and impact upon the job profile of the clinical statistician. One advantage of a joint curriculum would be that core training components form an integral (and not optional) part of local doctoral requirements, as currently the case within IDEAS. Furthermore, a standardisation of the requirements for the thesis and courses, ie, in terms of European Credit Transfer System (ECTS) points, would also facilitate further mobility of ESRs across countries. We envisage that students would be able to enrol at any of the participating academic institutions. Once enrolled, their primary place of research would be at that institution or at a nonacademic partner. However, all participating ESRs will undertake the same training and processes (in terms of monitoring as well as degree requirements) irrespective of their primary site of work. This has the immediate advantage that all graduates from the programme would benefit from an established international professional network that spans both academia and industry. At the same time, this programme can be seen as a specialist certification that graduates have an excellent working knowledge of the statistics of drug development.

During the preparation of their thesis, a student should be cosupervised by an academic and industry researcher. The PhD thesis should ideally consist of methodological work, which should be of a standard suitable for publication in high‐quality peer‐reviewed journals, supplemented by an overarching introduction and discussion. Ideally, at least one paper should have been accepted by the time of submission, although it is recognised that these timelines are not within the control of the student. Therefore, the thesis should be assessed by 2 independent experts in the field and be defended at an oral examination (similar to the viva voce examination in the United Kingdom).

We believe that such a joint curriculum must require students to have spent a minimum of 3 months at a pharmaceutical company or regulator and have worked with at least 2 different organisations located in different countries. Moreover, we believe that it is essential that students are taught the areas detailed in Table 4. These courses, which we feel are a minimum requirement, will certainly need to be supplemented by training in areas relevant to the researcher's project (eg, survival analysis for a project related to this topic). The compulsory training should be organised as training blocks, and some additional courses should be offered online (with assessed components).

**Table 4:** Formal training for a curriculum in statistics in drug development

Overview of the drug development process Relevance: Fundamental understanding of drug development Duration: 2 d (16 h) F2F/4 h online Topics: General overview on drug development (safety, preclinic, phases I‐IV, …), different study designs (such as parallel vs crossover studies and superiority vs equivalence), sample size, determination, regulatory aspects (eg, relevant guidelines and clinical trial regulation), quantitative methods for benefit‐risk assessment, and pharmacovigilance Skills acquired: To adequately design studies and drug development programme appreciating the legal requirements
Developing a clinical trial protocol Relevance: Core to any clinical study is the protocol Duration: Half day (4 h) F2F Topics: Role of a clinical trial protocol, regulation, elements (background, study objectives, study design, endpoints, eligibility criteria, …), and structure of a protocol. Difference between study protocol and separate statistical analysis plan, key elements for outlining randomisation, and statistical analysis in protocol. Case studies of good and bad protocols. Skills acquired: To adequately describe the clinical trial and the planned analysis. Being able to write especially the statistical parts for a clinical trial protocol.
Statistical methods for research and preclinical development Relevance: Understanding of challenges and statistical solutions in the area of research and preclinical development Duration: Half day (4 h) F2F Topics: Introduction to statistics in research and preclinical development, reproducibility, complex statistical techniques in research, statistics in preclinical safety, Dunnett and William test, Bayes approach to dose‐response characterisation, and carcinogenicity Skills acquired: Understanding challenges in statistical consultation and evaluation of experiments in research and preclinical safety and basic understanding of common approaches
Dose‐finding Relevance: Dose‐finding is one of the major tasks in drug development Duration: 2 d (16 h) F2F Topics: Dose‐finding in drug development (overview), sequential phase I dose‐finding trials to identify an optimal dose, model‐based Bayesian and likelihood methods for phase I trials, parallel group adaptive and nonadaptive phase II dose‐finding trials, and MCP‐Mod methodology Skills acquired: Understanding the dose‐finding problem inside and outside oncology. Practical design and analysis of dose‐finding trials in phases I and II.
Advanced design Relevance: To make efficient use of incoming and external data for decision‐making Duration: 3 d (24 h) F2F Topics: Randomisation and stratification, optimal designs, trial designs allowing early stopping for futility or efficacy (ie, group sequential design), and trials that can be altered based on accumulating data (eg, sample size reassessment and treatment or subgroup selection). Statistical methodology will include methods with pre‐fixed rules, combination tests, conditional error rates and worst‐case adjustments and extension to more complex trial designs addressing multiple objectives (eg, closed test procedures). Skills acquired: Optimal design, understanding of frequentist properties, and multiple testing procedures.
Pharmacological modelling Relevance: Emphasis is on the estimation of physiologically relevant information characterising the subject dependent drug exposure and relating it to a (un)wanted pharmacodynamical effect Duration: 1 d (8 h) F2F Topics: Pharmacometrics being the field closest related to biostatistics, the communalities and differences between the 2 expertises are highlighted. Noncompartmental methods are illustrated and explained. These are extended to compartmental pharmacokinetic models and pharmacokinetic models with differential equations. The connection with pharmacodynamics is explored, with the Emax model for physiological effects derived from first principles, and the importance of pharmacological modelling to drug development in general is illustrated. More broadly, the course serves as an introduction to non‐linear mixed effects modelling and its implementation in SAS Skills acquired: Understanding of pharmacokinetics‐ and physiology‐inspired modelling. On successful completion of the course, students should be able to fit non‐linear mixed‐effect models in SAS using proc nlmixed and interpret the SAS output.
Advanced computational skills including efficient computing Relevance: Efficient use of computing is vital to conduct simulation studies or implement complex statistical techniques. Duration: Half a day (4 h) F2F/16 h online Topics: Parallel computing in R including simple approaches to making code parallelisable and the use of the *parallel* package in R. Dynamic report creation via the *knitr* package and *RMarkdown*. Guided‐user interface‐based data visualisation using shiny. Skills acquired: Ability to use multicore processors to speed up simulations. Reproducible research methods
Statistical methods for evidence synthesis Relevance: To understand the statistical principles of systematic reviews and meta‐analysis and become able to support such reviews from the statistical point of view Duration: Half a day (4 h) F2F/4 h online Topics: Overview of statistical evidence synthesis methodology, in particular, fixed‐effects and random‐effects approaches, measures of heterogeneity, meta‐regression analysis, and brief introduction to network meta‐analysis Skills acquired: Understanding the statistical principles for meta‐analyses and systematic reviews
Statistical inference and multiple testing: Relevance: Enabling valid conclusions Duration: 8 h online Topics: Frequentist and Bayesian approaches to address specific research questions, prespecification vs exploration. Regression to the mean, decision theory, error rates in the context of testing single and multiple hypotheses, resampling‐based methods, and closed testing. Skills acquired: An in‐depth understanding of statistical inference to design and analyse clinical trials
Statistical learning Relevance: Analysis of large biomedical data set Duration: 2 d F2F/4 h online Topics: Interdisciplinary applications of machine learning and other data science techniques, for example, in electronic health records, high throughput screening, genomics, pharmacovigilance covering methods as regression and classification, model selection, resampling methods, large‐scale multiple testing, and clustering. Skills acquired: Knowledge of the concepts of statistical learning; understanding of a select set of statistical learning approaches and their application in R.

Additionally, we are convinced that it is paramount that each student should receive training in the transferable and generic skills given in Table 5 to thrive in their future roles.

**Table 5:** Transferable and generic skills in joint curriculum

Working in a culturally diverse environment Relevance: To sensitise the researchers about cultural differences Duration: 2 h F2F Topics: Key experiences with cultural differences of assumptions, values, and ethics. Skills acquired: An appreciation for diversity and some ideas to avoid misunderstanding.
Ethics in research Relevance: To raise awareness about the ethical dimension of research Duration: 4 h F2F Topics: What ethical issues should be considered in planning and conducting research? When does the research require ethical approval? What ethical norms are to be adhered to when publishing research results? Algorithmic responsibility. Skills acquired: Understanding ethical considerations relevant to conducting research.
Presentation skills Relevance: To improve presentation skills Duration: 1 d (8 h) F2F Topics: Planning and preparing a presentation, different presentation techniques, body language, and interacting with audience, managing nervousness, and using voice effectively. Skills acquired: To plan and deliver confident, effective presentations
Planning and managing a project Relevance: To develop successful strategies for conducting a project successfully Duration: Half a day (4 h) F2F Topics: Planning, organising, and managing a project: Breaking projects down into “bitesized chunks,” developing realistic timelines, and prioritisation of tasks. Skills acquired: The ability to effectively plan and organise a project and monitor the projects progress
How to make a case for funding of a research project Relevance: Do's and don'ts when applying for research funding Duration: Half a day (4 h) F2F Topics: Funding application work flow, funding schemes in Europe, differences between public and industry funding, and case studies. Skills acquired: Understanding the process to gain funding and tips for successful applications
Entrepreneurial skills for a start‐up/small company Relevance: Enhance future career options Duration: Half a day (4 h) F2F Topics: (i) How to create competitive business advantages, (ii) planning and controlling (business and financial planning, risk assessment, intellectual property right issues, …), (iii) how to reach clients (advertisement in print, social media, websites, presence at conferences, and organisation of events), and (iv) customer acquisition (efficient presentations, contracting, price negotiations, and developing customer loyalty). Skills acquired: Ability to set up projects and enterprises.
Multidisciplinary collaborations Relevance: Enabling successful collaboration in teams Duration: Half a day (4 h) F2F Topics: Negotiations skills, layman's presentations, eg, explaining statistical terms to a nonstatistical partner, input to nonstatistical discussions, eg, when developing a study protocol Skills acquired: Ability to communicate statistical concepts to nonexperts; improved communication skills.
Reproducible research Relevance: When research fails, it can be because of mistakes made early in its development and one way to identify these mistakes is to check if the early research findings are reproducible. Statisticians are often key collaborators in research and typically are involved in the analysis and interpretation of data. It is therefore important that these analyses are demonstrably reproducible and statisticians will need to learn the skills necessary to do this. This course will describe and illustrate the tools available in RStudio to help make research reproducible. Duration: Half a day (4 hr) F2F Topics: Define reproducible research, highlight importance of reproducible research, and become familiar with tools (such as RMarkup) to help make research reproducible. Skills acquired: Ability to conduct reproducible research; knowledge of specialised tools for reproducible research.

With such a programme, students would be well placed to start work not only as statisticians within the pharmaceutical industry but also as academic researchers or within regulatory agencies. Having a harmonised European PhD curriculum in place might also help to define what educational and professional knowledge is needed to be a “statistician” in drug development.

## 7 DISCUSSION

While the early drug development phases are increasingly moving into the focus of applied pharmaceutical research, this seems to be less so in the education of statistical sciences. The desire for novel statistical methodology is no longer confined to late‐stage development, as the demand for quantitative methods to support decisions in the transition phases surges.

The IDEAS network is a first step towards harmonising the academic education of PhD students with an interest in early‐phase studies. It brings together all of the key skills required for a career as a research scientist in academia, the pharmaceutical industry, CROs or regulatory agency, and for entrepreneurs embarking on statistical start‐up companies.

The network provides access to European key opinion leaders, clinical trials experts, and other stakeholders in the public and private sectors. The extensive course programme ensures the development of statistical expertise in designing, conducting, analysing, and assessing innovative early‐phase trials. Statistical courses provide a well‐founded basis in traditional topics relevant for early development to ensure that graduates have a broad and in‐depth competence on state‐of‐the‐art methods. As the consortium is composed of experts in different fields, including novel methodology into the proposed curriculum and training courses is straightforward. This gives the graduates a head start, having learnt new methods directly from their originators and early adopters in industry.

The specific courses focusing on project planning as well as the work on the individual research projects will provide ESRS with the skills to independently develop research plans, write grant, or project proposals, which are essential for a successful career as a research scientist in both the public and private sectors.

Dedicated training courses and regular progress presentations at the summer schools, workshops, secondments, and research visits ensure that communication and presentation skills are acquired. Skills in scientific writing in technical, medical journals as well as for the public will be developed. Focus is given to the ability to present complex, technical matters to nonstatisticians such as the medical community and general public. This is to ensure a smooth translation of methodological work from the statistical to the medical community. Transnational, cross‐sectorial secondments will ensure that the researchers understand the needs and perspectives of different stakeholders and will enhance their multicultural competence to initiate and facilitate international research collaborations.

There is an increasing demand for biostatisticians with profound expertise in medical studies, especially in the highly regulated field of drug development so that the graduates of IDEAS will be in high demand from a range of different employers: They can have a career path as a scientific researcher at a university, a statistician/methodologist within the pharmaceutical industry, or a statistician within a clinical trial unit. Similarly, regulatory and health technology assessment bodies have an increasing demand for qualified statisticians to assess increasingly complex trial designs and interpret the resulting data. The largest employers of biostatisticians are based on the pharmaceutical industry and CROs, where biostatisticians are in constant high demand to work on clinical projects level as well as in methodology groups.

We believe that IDEAS is a step forward in the training of statisticians working in drug development. The IDEAS network does, however, have 2 key shortcomings. Firstly, it is focused on early drug development, while the need for similarly trained researchers covers the entire drug development process. Secondly, IDEAS is, at the moment, a single initiative on a relatively small scale (14 researchers will be trained through the programme) for a limited time (from 2015 to 2018). To overcome this, our vision is to build on the principles of IDEAS to develop a joint, harmonised PhD level programme that is cross‐sectorial, cross‐national for statisticians working in drug development. Having in place a harmonised European PhD curriculum might also help to define what educational and professional background constitutes a “statistician” for drug development. The key to achieving this vision is continued and even strengthened interaction between industry and academia and a close collaboration between academic institutions across Europe and beyond.

In our proposal, we have considered student to be full‐time although there are a number of students with in an interest in pharmaceutical statistics that study part‐time. Many of these students work at a pharmaceutical company or CRO alongside undertaking their PhD studies. The main challenges in accommodating such part‐time students would be the integration of these students in the cohort of full‐time students and the requirement to take part in (intensive) training weeks, which might conflict with other work commitments.

## FUNDING

This project has received funding from the European Unions Horizon 2020 research and innovation programme under the Marie Sklodowska‐Curie grant agreement no. 633567 and by the Swiss State Secretariat for Education, Research, and Innovation (SERI) under contract number 999754557. The opinions expressed and arguments used herein do not necessarily reflect the official views of the Swiss Government. http://www.ideas-itn.eu/

## Supporting information


**Table S1** Compulsory and optional modules on example MSc programmes in Statistics in Europe

**Table S2** Compulsory and optional modules on MSc programmes in Statistics in the UK

## APPENDIX A.

### A..1

**Table A1:** Summer school (time is given in hours [h] and days [d], whereby a full day is equivalent to 8 h)

	SUMMERSCHOOL (S)			
	S1 (Kick‐Off)	S2	S3	S4
Specialised training				
Selected research presentations/lectures by renown researchers in the field	2 h	1 h		
A regulatory view of drug development	2 h			
An introduction to drug development	1.5 d			
Ethics in research (general, clinical trials, and regulations)	4 h			
Statistical computing (parallel computing in R; shiny—interactive data visualisation)		0.5 d		
Pharmacological modelling		1 d		
Practical workshop on e‐learning course “Genomics”			1 d	
Adaptive methods for dose‐finding			1 d	
Adaptive clinical trials				1 d
Data and safety monitoring board				0.5 d
Individually supervised research projects				
Short intro to individually supervised research projects	3 h			
ESR update on research projects		1 d	1 d	0.5 d
Clinical advisor experiences		1 h	1 h	
Training on transferable skills				
Working in a culturally diverse environment	2 h			
The art of giving presentations – Soft skills training		0.5 d		
Planning and managing a project		0.5 d		
How to write a successful job application			0.5 d	
How to successfully obtain research funding			0.5 d	
How to write a business plan for a statistical consulting company			0.5 d	
Developing entrepreneurial skills—how to start your own statistical company				0.5 d
Working with medical collaborators				0.5 d
Team building activities				
	1.5 d	1 d	0.5 d	0.5 d
All other business				
Overview of project by coordinator	1 h			
Partner introductions	1 h			
Administrative board meeting	0.5 d	1 h	1.5 h	1.5 h

**Table A2:** E‐learning courses offered

Year	Course Title
1	Computational skills in statistics
2	Genomics: technologies and data analyses
3	Multiple testing
