# Supplementary material for: A proposal for a new PhD level curriculum on quantitative methods for drug development
Source: Pharm Stat. 2018 Jul 9;17(5):593–606. doi: 10.1002/pst.1873 (PMC6174936; doi:10.1002/pst.1873)
Supplement: Supplementary file 1 — Table S1 Compulsory and optional modules on example MSc programmes in Statistics in Europe [file PST-17-593-s001.docx]

**Supplementary material to**

“**A proposal for a new PhD level curriculum on quantitative methods for drug development”**

Jaki T^1^, Gordon A^1^, Forster P^1^, Bijnens L^2^, Bornkamp B^3^, Brannath W^4^, Fontana R^5^, Gasparini M^5^, Hampson LV^1,6^, Jacobs T^2^, Jones B^3^, Paoletti X^7^, Posch M^8^, Titman A^1^, Vonk R^9^, Koenig F^8,*^

Table S1 Compulsory and optional modules on example MSc programmes in Statistics in Europe

| **Country** | **Institution** |  | **Elective Modules** |
| --- | --- | --- | --- |
|  |  | **Compulsory Modules** |  |
| Austria | [Universitat Wien](http://slw.univie.ac.at/studieren/studienangebot/masterstudien/statistik-master/) | Probability Theory and Asymptotic Statistics  Stochastics  Statistical case studies  Applied Optimisation  Biometry | (Taken from other Master's programmes) |
| Belgium | [Hasselt University](https://www.uhasselt.be/studyguide?n=3&i=001) | Concepts of Bayesian Inference  Learning from Data  Concepts of Probability and Statistics  Software and Data Management  Linear Models  Nonparametric Methods  Sampling Theory  Generalised Linear Models  Multivariate and Hierarchical Data  Bayesian Data Analysis  Survival Data Analysis  Topics in Advanced Modelling Techniques  Principles of Statistical Inference  Longitudinal Data Analysis  Clinical Trials | Concepts of Bioinformatics  Concepts of Epidemiology  Statistical and Computational Methods for Integrated Analysis  Analysis of High Dimensional Omics Data  Spatial Epidemiology  Modelling Infectious Diseases  Data Mining  Advanced Methods for Genomics |
| Denmark | [University of Copenhagen](http://studies.ku.dk/masters/statistics/) | Discrete Models  Graphical Models  Regression  Project in Statistics | Advanced Probability  Computational Statistics  Stochastic Models and Inference for Genetic Data  Survival Analysis  Causality  Modern Topics in Statistics  Numerical Optimisation |
| France | [Université Pierre et Marie Curie - Paris 6](http://www.master.ufrmath.upmc.fr/) | Statistics applied to clinical research  Epidemiology  Statistics applied to biology | Advanced clinical research  Censored data  Bayesian analysis for clinical research  Introduction to genetics  Statistics and genome  Experimental design |
| Germany | [University Dortmund](https://www.statistik.tu-dortmund.de/fileadmin/user_upload/Studium/Ordnungen/Pruefungs-_und_Studienordnungen/Pruefungsordnung_Master_Statistik_2015.pdf) | Probability theory  Decision theory  Advanced design of experiments  Stochastic processes  Sampling theory | Survival analysis  Bioassays  Clinical trials  Epidemiology  Statistical genetics  Biometrical methods in drug development  Gene network modelling  Meta analysis  Adaptive clinical trials |
| Germany | [Universitat Bremen](https://www.dbs.uni-bremen.de/studienangebot/studien-berufsfelder/natur-umwelt/detail/study/medical-biometry-biostatistics-master-1/) | Biometric Methods  Statistical Modelling  Data Management  Statistical Programming  Basis of Epidemiology  Clinical Studies and Ethics  Clinical Studies, Laws & Guidelines  Medical Basics  Internal Medicine & Pharmacology | General courses in Application of Biometric Methodology |
| Greece | [University of Athens](http://biostatistics.med.uoa.gr/index.php/study-guide/) | Introduction to Probabilities  Introduction to Epidemiology  Introduction to Statistics and Biostatistics  Regression and Dispersion Analysis  Generalised Linear Models  Survival Analysis  Statistical Methods in Epidemiology  Research Methodology | Nonparametric Statistics  Data Management  Clinical Tests  Sampling Methods  Bayesian Inference  Multivariate Analysis  Meta-Analysis  Reported Measurement Data Analysis  Statistical and Graphics Packages |
| Ireland | [University College Dublin](https://sisweb.ucd.ie/usis/!W_HU_MENU.P_PUBLISH?p_tag=PROG&MAJR=T020) | Technical Communication  Mathematical Statistics | Numerical Algorithms  Uncertainty Quantification  Data Mining  Survival Models  Categorical Dada Analysis  Multivariate Analysis  Bayesian Analysis  Applied Statistical Modelling  Regression Methods  Stochastic Models |
| Italy | [University of Bologna](http://corsi.unibo.it/2Cycle/StatisticalSciences/Pages/course-structure-and-teaching-activities.aspx) | Fundamental Concepts of Statistics  Statistical Models and Applications  Stochastic Process and Advanced Time Series  Nonparametric Statistics  Statistical Methods for Clinical Research  Statistical Software  Analysis of Categorical Data  Bayesian Inference  Advanced Survival Analysis  Modern Statistics and Big Data Analysis  Latent Variable Models | Survey Sampling  Discrete Mathematics  Differential Equations  Computational Human Genomics  Systems and Algorithms for Data Science |
| Poland | [Wroclaw University](http://wmat.pwr.edu.pl/studenci/studia/programy-studiow/studia-ii-stopnia/matematyka) | Stochastic Processes  Mathematical Statistics  Real and Complex Analysis  Partial Differential Equations  Functional Analysis and Topology  Special Functions  Basics of Quantum Mechanics | Nonparametric Statistics  Analysis of Time Series  Stochastic Contract Models  Analysis of Survey Data  Applied Statistics  Testing Hypotheses  Optimal Sequential Procedures  Non-Linear Analytical Methods  Theory of Estimation |
| Spain | [Universidade de Santiago de Compostela](http://www.usc.es/masteres/en/masters/science/statistical-techniques) | Models of Regression  Exploratory Analysis of Data  Complete Linear Programming  Models of Probability  Applied Statistics | Stochastic Processes  Nonparametric Statistics  Sampling  Time Series  Design and Analysis of Experiments  Statistical Simulation  Multivariate Analysis  Spatial Statistics  Reliability and Biometric Models |
| Switzerland | [University of Geneva](https://www.unige.ch/gsem/en/programs/masters/statistics/) | Analytics Consulting  Generalised Linear and Additive Models  Mixed Linear Models  Multivariate Analysis  Sampling Techniques  Time Series | Advanced Statistical Inference  Introduction to Biostatistics  Multivariate Models  Structural Equation Modelling  Data Mining  Statistical Estimation |
| United Kingdom | [Lancaster University](http://www.lancaster.ac.uk/maths/) | Bayesian Inference  Likelihood Inference  Statistics in Practice  Generalised Linear Models  Computational Intensive Methods | Clinical Trials  Pharmacological Modelling  Adaptive and Bayesian methods in clinical research  Survival and Event History Analysis  Genomics: technologies and data analysis  Principles of Epidemiology |

Table S2 Compulsory and optional modules on MSc programmes in Statistics in the UK

| **University** | **Compulsory modules** | **Relevant optional modules** |
| --- | --- | --- |
| [Imperial College London](http://www.imperial.ac.uk/natural-sciences/departments/mathematics/study/admissions/postgraduate/msc/statistics/) | Probability for Statistics  Fundamentals of Statistical Inference  Applied Statistics  Computational Statistics | Medical Statistics  Statistical Bioinformatics and Genetics |
| [University of Nottingham](https://www.nottingham.ac.uk/pgstudy/courses/mathematical-sciences/statistics-msc.aspx) | Fundamentals of Statistics  Medical Statistics |  |
| [University of Warwick](http://www2.warwick.ac.uk/fac/sci/statistics/postgrad/msc/) | Statistical Methods  An Introduction to Statistical Practice | Medical Statistics with Advanced Topics  Statistical Genetics with Advanced Topic |
| [University College London](https://www.ucl.ac.uk/statistics/prospective-postgraduates/msc-medical-statistics) | Foundations Course  Statistical Models & Data Analysis  Statistical Computing  Applied Bayesian Methods  Statistical Inference  Medical Statistics I  Medical Statistics II | Epidemiology  Bayesian Methods in Health Economics  Statistics for interpreting Genetic Data |
| [University of Kent](http://www.kent.ac.uk/courses/postgraduate/166/statistics#!structure) | Computational Statistics  Probability and Classical Inference  Advanced Regression Modelling  Bayesian Statistics  Principles of Data Collection  Practical Statistics and Computing | Stochastic Models in Ecology and Medicine |
| [Lancaster University](http://www.lancaster.ac.uk/maths/postgraduate/courses/statistics-msc/#coursestructure) | Bayesian Inference  Likelihood Inference  Statistics in Practice  Generalised Linear Models  Computational Intensive Methods | Clinical Trials  Pharmacological Modelling  Adaptive and Bayesian methods in clinical research  Survival and Event History Analysis  Genomics: technologies and data analysis  Principles of Epidemiology |
| [University of Leeds](https://www.maths.leeds.ac.uk/postgraduate-taught/courses/msc-medical-statistics.html) | Introduction to Clinical Trials  Core Epidemiology  Introduction to Modelling  Statistical Computing | Advanced Epidemiological techniques  Generalised Linear Models and Survival - Analysis  Statistics and DNA  Advanced epidemiological techniques |
| [University of Sheffield](http://www.sheffield.ac.uk/postgraduate/taught/courses/purescience/mathstats/statistics-medical-applications-msc) | Data Analysis  Statistical Laboratory  Linear Models  Epidemiological Methods and Time Series  Bayesian Inference and Further Clinical Trials  Sampling, Design and Medical Statistics |  |
| [London School of Hygiene & Tropical Medicine](http://www.lshtm.ac.uk/study/masters/msms.html) | Basic Epidemiology  Clinical Trials  Foundations of Medical Statistics  Introduction to Statistical Computing  Robust Statistical Methods  Generalised Linear Models  Statistical Methods in Epidemiology  Survival Analysis and Bayesian Statistics | Advanced Statistical Methods in Epidemiology  Epidemiology of Non-Communicable Diseases  Modelling & the Dynamics of Infectious Diseases  Social Epidemiology |
| [University of Southampton](http://www.southampton.ac.uk/maths/postgraduate/taught_courses/diploma_msc_in_statistics_with_applications_in_medicine.page#modules) | Statistical Theory and Linear Models  Statistical Computing  Design of Experiments  Clinical Trials  Epidemiological Methods  Generalised Linear Models  Survival Analysis  Research Skills | Statistical Genetics |
| [University of Leicester](https://le.ac.uk/courses/medical-statistics-msc) | Fundamentals of Medical Statistics  Statistical Modelling  Computationally Intensive Methods  Advanced Statistical Modelling  Clinical Trials  Epidemiology | Further Topics in Medical Statistics  Genetic Epidemiology  Health Technology Assessment |
| [University of Glasgow](http://www.gla.ac.uk/postgraduate/taught/biostatistics/#/programmestructure) | Bayesian statistics  Biostatistics  Generalised linear models  Introduction to R programming  Probability 1  Regression models  Statistical inference 1  Statistics project and dissertation | Design of Experiments  Statistical Genetics |
| [University of Strathclyde](http://www.strath.ac.uk/courses/postgraduatetaught/appliedstatisticsinhealthsciences/) | Foundations of Probability & Statistics  Data Analytics in R  Applied Statistical Modelling  Medical Statistics  Bayesian Spatial Statistics  Effective Statistical Consultancy  Risk Analysis  Survey Design & Analysis |  |
| [University of Edinburgh](http://msc.maths.ed.ac.uk/or/index) | Statistical Regression Models  Methodology, Modelling and Consulting Skills  Computing for Statistics  Stochastic Modelling  Likelihood and Generalised Linear Models  Fundamentals of Optimization  Fundamentals of Operational Research  Simulation | Clinical Trials  Genetic Epidemiology  Stochastic Models in Biology  Analysis of Survival Data |
