# Supplementary material for: A proposal for a new PhD level curriculum on quantitative methods for drug development
Source: Pharm Stat. 2018 Jul 9;17(5):593–606. doi: 10.1002/pst.1873 (PMC6174936; doi:10.1002/pst.1873)
Supplement: Supplementary file 2 — Table S2 Compulsory and optional modules on MSc programmes in Statistics in the UK [file PST-17-593-s002.pdf]

Supplementary material to

"A proposal for a new PhD level curriculum on quantitative methods for drug development"

Jaki T[1], Gordon A[1], Forster P[1], Bijnens L[2], Bornkamp B[3], Brannath W[4], Fontana R[5], Gasparini M[5], Hampson LV[1,6], Jacobs T[2], Jones B[3], Paoletti X[7], Posch M[8], Titman A[1], Vonk R[9], Koenig F[8,*]

Table S1 Compulsory and optional modules on example MSc programmes in Statistics in Europe

Country	Institution	Compulsory Modules	Elective Modules
Austria	Universitat Wien	Probability Theory and Asymptotic Statistics<br>Stochastics<br>Statistical case studies<br>Applied Optimisation<br>Biometry	(Taken from other Master's programmes)
Belgium	Hasselt University	Concepts of Bayesian Inference<br>Learning from Data<br>Concepts of Probability and Statistics<br>Software and Data Management<br>Linear Models<br>Nonparametric Methods<br>Sampling Theory<br>Generalised Linear Models<br>Multivariate and Hierarchical Data<br>Bayesian Data Analysis<br>Survival Data Analysis<br>Topics in Advanced Modelling Techniques<br>Principles of Statistical Inference<br>Longitudinal Data Analysis<br>Clinical Trials	Concepts of Bioinformatics<br>Concepts of Epidemiology<br>Statistical and Computational Methods for Integrated Analysis<br>Analysis of High Dimensional Omics Data<br>Spatial Epidemiology<br>Modelling Infectious Diseases<br>Data Mining<br>Advanced Methods for Genomics
Denmark	University of Copenhagen	Discrete Models<br>Graphical Models<br>Regression<br>Project in Statistics	Advanced Probability<br>Computational Statistics<br>Stochastic Models and Inference for Genetic Data<br>Survival Analysis<br>Causality<br>Modern Topics in Statistics<br>Numerical Optimisation
France	Université Pierre et Marie Curie - Paris 6	Statistics applied to clinical research<br>Epidemiology<br>Statistics applied to biology	Advanced clinical research<br>Censored data<br>Bayesian analysis for clinical research<br>Introduction to genetics<br>Statistics and genome<br>Experimental design
Germany	University Dortmund	Probability theory<br>Decision theory<br>Advanced design of experiments<br>Stochastic processes<br>Sampling theory	Survival analysis<br>Bioassays<br>Clinical trials<br>Epidemiology<br>Statistical genetics<br>Biometrical methods in drug development<br>Gene network modelling<br>Meta analysis<br>Adaptive clinical trials
Germany	Universitat Bremen	Biometric Methods<br>Statistical Modelling<br>Data Management<br>Statistical Programming<br>Basis of Epidemiology<br>Clinical Studies and Ethics<br>Clinical Studies, Laws & Guidelines<br>Medical Basics<br>Internal Medicine & Pharmacology	General courses in Application of Biometric Methodology
Greece	University of Athens	Introduction to Probabilities<br>Introduction to Epidemiology<br>Introduction to Statistics and Biostatistics<br>Regression and Dispersion Analysis<br>Generalised Linear Models	Nonparametric Statistics<br>Data Management<br>Clinical Tests<br>Sampling Methods<br>Bayesian Inference

		Survival Analysis<br>Statistical Methods in Epidemiology<br>Research Methodology	Multivariate Analysis<br>Meta-Analysis<br>Reported Measurement Data Analysis<br>Statistical and Graphics Packages
Ireland	University College Dublin	Technical Communication<br>Mathematical Statistics	Numerical Algorithms<br>Uncertainty Quantification<br>Data Mining<br>Survival Models<br>Categorical Dada Analysis<br>Multivariate Analysis<br>Bayesian Analysis<br>Applied Statistical Modelling<br>Regression Methods<br>Stochastic Models
Italy	University of Bologna	Fundamental Concepts of Statistics<br>Statistical Models and Applications<br>Stochastic Process and Advanced Time Series<br>Nonparametric Statistics<br>Statistical Methods for Clinical Research<br>Statistical Software<br>Analysis of Categorical Data<br>Bayesian Inference<br>Advanced Survival Analysis<br>Modern Statistics and Big Data Analysis<br>Latent Variable Models	Survey Sampling<br>Discrete Mathematics<br>Differential Equations<br>Computational Human Genomics<br>Systems and Algorithms for Data Science
Poland	Wroclaw University	Stochastic Processes<br>Mathematical Statistics<br>Real and Complex Analysis<br>Partial Differential Equations<br>Functional Analysis and Topology<br>Special Functions<br>Basics of Quantum Mechanics	Nonparametric Statistics<br>Analysis of Time Series<br>Stochastic Contract Models<br>Analysis of Survey Data<br>Applied Statistics<br>Testing Hypotheses<br>Optimal Sequential Procedures<br>Non-Linear Analytical Methods<br>Theory of Estimation
Spain	Universidade de Santiago de Compostela	Models of Regression<br>Exploratory Analysis of Data<br>Complete Linear Programming<br>Models of Probability<br>Applied Statistics	Stochastic Processes<br>Nonparametric Statistics<br>Sampling<br>Time Series<br>Design and Analysis of Experiments<br>Statistical Simulation<br>Multivariate Analysis<br>Spatial Statistics<br>Reliability and Biometric Models
Switzerland	University of Geneva	Analytics Consulting<br>Generalised Linear and Additive Models<br>Mixed Linear Models<br>Multivariate Analysis<br>Sampling Techniques<br>Time Series	Advanced Statistical Inference<br>Introduction to Biostatistics<br>Multivariate Models<br>Structural Equation Modelling<br>Data Mining<br>Statistical Estimation
United Kingdom	Lancaster University	Bayesian Inference<br>Likelihood Inference<br>Statistics in Practice<br>Generalised Linear Models<br>Computational Intensive Methods	Clinical Trials<br>Pharmacological Modelling<br>Adaptive and Bayesian methods in clinical research<br>Survival and Event History Analysis<br>Genomics: technologies and data analysis<br>Principles of Epidemiology

Table S2 Compulsory and optional modules on MSc programmes in Statistics in the UK

University	Compulsory modules	Relevant optional modules
Imperial College London	Probability for Statistics<br>Fundamentals of Statistical Inference<br>Applied Statistics<br>Computational Statistics	Medical Statistics<br>Statistical Bioinformatics and Genetics
University of Nottingham	Fundamentals of Statistics<br>Medical Statistics	
University of Warwick	Statistical Methods<br>An Introduction to Statistical Practice	Medical Statistics with Advanced Topics<br>Statistical Genetics with Advanced Topic
University College London	Foundations Course<br>Statistical Models & Data Analysis	Epidemiology<br>Bayesian Methods in Health Economics

	Statistical Computing<br>Applied Bayesian Methods<br>Statistical Inference<br>Medical Statistics I<br>Medical Statistics II	Statistics for interpreting Genetic Data
University of Kent	Computational Statistics<br>Probability and Classical Inference<br>Advanced Regression Modelling<br>Bayesian Statistics<br>Principles of Data Collection<br>Practical Statistics and Computing	Stochastic Models in Ecology and Medicine
Lancaster University	Bayesian Inference<br>Likelihood Inference<br>Statistics in Practice<br>Generalised Linear Models<br>Computational Intensive Methods	Clinical Trials<br>Pharmacological Modelling<br>Adaptive and Bayesian methods in clinical research<br>Survival and Event History Analysis<br>Genomics: technologies and data analysis<br>Principles of Epidemiology
University of Leeds	Introduction to Clinical Trials<br>Core Epidemiology<br>Introduction to Modelling<br>Statistical Computing	Advanced Epidemiological techniques<br>Generalised Linear Models and Survival - Analysis<br>Statistics and DNA<br>Advanced epidemiological techniques
University of Sheffield	Data Analysis<br>Statistical Laboratory<br>Linear Models<br>Epidemiological Methods and Time Series<br>Bayesian Inference and Further Clinical Trials<br>Sampling, Design and Medical Statistics	
London School of Hygiene & Tropical Medicine	Basic Epidemiology<br>Clinical Trials<br>Foundations of Medical Statistics<br>Introduction to Statistical Computing<br>Robust Statistical Methods<br>Generalised Linear Models<br>Statistical Methods in Epidemiology<br>Survival Analysis and Bayesian Statistics	Advanced Statistical Methods in Epidemiology<br>Epidemiology of Non-Communicable Diseases<br>Modelling & the Dynamics of Infectious Diseases<br>Social Epidemiology
University of Southampton	Statistical Theory and Linear Models<br>Statistical Computing<br>Design of Experiments<br>Clinical Trials<br>Epidemiological Methods<br>Generalised Linear Models<br>Survival Analysis<br>Research Skills	Statistical Genetics
University of Leicester	Fundamentals of Medical Statistics<br>Statistical Modelling<br>Computationally Intensive Methods<br>Advanced Statistical Modelling<br>Clinical Trials<br>Epidemiology	Further Topics in Medical Statistics<br>Genetic Epidemiology<br>Health Technology Assessment
University of Glasgow	Bayesian statistics<br>Biostatistics<br>Generalised linear models<br>Introduction to R programming<br>Probability 1<br>Regression models<br>Statistical inference 1<br>Statistics project and dissertation	Design of Experiments<br>Statistical Genetics
University of Strathclyde	Foundations of Probability & Statistics<br>Data Analytics in R<br>Applied Statistical Modelling<br>Medical Statistics<br>Bayesian Spatial Statistics<br>Effective Statistical Consultancy<br>Risk Analysis<br>Survey Design & Analysis	
University of Edinburgh	Statistical Regression Models<br>Methodology, Modelling and Consulting Skills<br>Computing for Statistics<br>Stochastic Modelling	Clinical Trials<br>Genetic Epidemiology<br>Stochastic Models in Biology<br>Analysis of Survival Data

	Likelihood and Generalised Linear Models<br>Fundamentals of Optimization<br>Fundamentals of Operational Research<br>Simulation	
